# Building clinical trial capacity to develop a new treatment for multidrug-resistant tuberculosis

**DOI:** 10.2471/BLT.15.154997

**Published:** 2015-11-17

**Authors:** Thelma Tupasi, Rajesh Gupta, Manfred Danilovits, Andra Cirule, Epifanio Sanchez-Garavito, Heping Xiao, Jose L Cabrera-Rivero, Dante E Vargas-Vasquez, Mengqiu Gao, Mohamed Awad, Leesa M Gentry, Lawrence J Geiter, Charles D Wells

**Affiliations:** a Tropical Disease Foundation, Makati City, Philippines.; b Otsuka Pharmaceutical Development and Commercialization, Inc., 2440 Research Boulevard, Rockville, Maryland 20850, United States of America.; c Tartu University Lung Hospital, Tartu, Estonia.; d State Agency of Tuberculosis and Lung Diseases, Riga, Latvia.; e Hospital Nacional Sergio E Bernales, Lima, Peru.; f Shanghai Pulmonary Hospital, Shanghai, China.; g Unidad de Investigacion, Hospital Nacional Daniel A Carrión, Lima, Peru.; h Hospital Nacional Hipólito Unanue, Lima, Peru.; i Beijing Chest Hospital, Beijing, China.; j Sadr Abassia Hospital, Cairo, Egypt.

## Abstract

**Problem:**

New drugs for infectious diseases often need to be evaluated in low-resource settings. While people working in such settings often provide high-quality care and perform operational research activities, they generally have less experience in conducting clinical trials designed for drug approval by stringent regulatory authorities.

**Approach:**

We carried out a capacity-building programme during a multi-centre randomized controlled trial of delamanid, a new drug for the treatment of multidrug-resistant tuberculosis. The programme included: (i) site identification and needs assessment; (ii) achieving International Conference on Harmonization – Good Clinical Practice (ICH-GCP) standards; (iii) establishing trial management; and (iv) increasing knowledge of global and local regulatory issues.

**Local setting:**

Trials were conducted at 17 sites in nine countries (China, Egypt, Estonia, Japan, Latvia, Peru, the Philippines, the Republic of Korea and the United States of America). Eight of the 10 sites in low-resource settings had no experience in conducting the requisite clinical trials.

**Relevant changes:**

Extensive capacity-building was done in all 10 sites. The programme resulted in improved local capacity in key areas such as trial design, data safety and monitoring, trial conduct and laboratory services.

**Lessons learnt:**

Clinical trials designed to generate data for regulatory approval require additional efforts beyond traditional research-capacity strengthening. Such capacity-building approaches provide an opportunity for product development partnerships to improve health systems beyond the direct conduct of the specific trial.

## Résumé

**Problème:**

Les nouveaux médicaments pour le traitement des maladies infectieuses doivent souvent être évalués dans des pays à faibles ressources. Même si le personnel qui travaille dans un tel contexte réalise souvent des soins et des activités de recherche opérationnelle de grande qualité, il est généralement moins expérimenté pour effectuer des essais cliniques en vue d'obtenir l'approbation des nouveaux médicaments par des autorités de réglementation très strictes.

**Approche:**

Nous avons mené un programme de renforcement des capacités à l'occasion d'un essai contrôlé randomisé multicentrique du delamanid; un nouveau médicament pour le traitement de la tuberculose multirésistante. Ce programme comprenait : (i) l'identification des sites et l'évaluation des besoins; (ii) l'application des normes de la Conférence internationale sur l'harmonisation relatives aux bonnes pratiques cliniques (normes ICH-GCP); (iii) la mise en œuvre d'une gestion des essais; et (iv) l'amélioration des connaissances sur les aspects réglementaires locaux et internationaux.

**Environnement local:**

Des essais ont été réalisés sur dix-sept sites, répartis dans neuf pays (Chine, Égypte, Estonie, États-Unis d'Amérique, Japon, Lettonie, Pérou, Philippines et République de Corée). Parmi les dix sites implantés dans des pays à faibles ressources, huit n'avaient aucune expérience dans la conduite des essais cliniques requis.

**Changements significatifs:**

Un important renforcement des capacités a été réalisé sur l'ensemble des dix sites. Ce programme s'est traduit par une amélioration des capacités locales dans des domaines clés comme la conception des essais, la sécurité et le contrôle des données, la conduite des essais et les services de laboratoire.

**Leçons tirées:**

Les essais cliniques conçus pour produire des données en vue d'obtenir l'approbation réglementaire nécessitent des efforts complémentaires, qui vont au-delà des activités traditionnelles de renforcement des capacités de recherche. Les approches de ce type visant à renforcer ces capacités offrent une opportunité de partenariat pour le développement de nouveaux produits en vue d'améliorer les systèmes de santé parallèlement à la réalisation directe des essais requis.

## Resumen

**Problema:**

Los nuevos fármacos para enfermedades infecciosas a menudo necesitan ser probados en emplazamientos con pocos recursos. Aunque los profesionales que trabajan en dichos emplazamientos ofrezcan una atención de alta calidad y lleven a cabo una actividad de investigación operativa, en general no tienen la experiencia suficiente para llevar a cabo los ensayos clínicos diseñados para aprobar fármacos según dictan los estrictos organismos de reglamentación.

**Enfoque:**

Se llevó a cabo un programa de fortalecimiento de la capacidad durante un ensayo multicéntrico controlado aleatorizado de delamanid, un nuevo fármaco para el tratamiento de la tuberculosis multirresistente. El programa incluía: (i) identificación del emplazamiento y evaluación de las necesidades; (ii) cumplimiento con las normas de buenas prácticas clínicas (BPC) de la Conferencia Internacional sobre Armonización (ICH); (iii) establecimiento de una gestión de los ensayos; y (iv) incremento del conocimiento de los aspectos regulatorios a nivel global y local.

**Marco regional:**

Los ensayos se llevaron a cabo en 17 emplazamientos de nueve países (China, Egipto, Estados Unidos de América, Estonia, Filipinas, Japón, Letonia, Perú y República de Corea). Ocho de los 10 lugares con pocos recursos no tenían experiencia alguna en cómo dirigir los ensayos clínicos necesarios.

**Cambios importantes:**

Se ha aumentado considerablemente la capacidad en todos los 10 emplazamientos. El programa se tradujo en una mejor capacidad local en aspectos clave como el diseño del ensayo, la seguridad y la supervisión de los datos, la realización de los ensayos y los servicios de los laboratorios.

**Lecciones aprendidas:**

Los ensayos clínicos diseñados para generar datos para una aprobación normativa requieren esfuerzos adicionales más allá del ya tradicional refuerzo de las capacidades de investigación. Dichos enfoques de aumento de la capacidad brindan una oportunidad para que las asociaciones para el desarrollo del producto mejoren los sistemas sanitarios, más allá de la realización directa del ensayo específico.

## ملخص

المشكلة غالبًا ما تحتاج الأدوية الجديدة لعلاج الأمراض المعدية إلى تقييمها في مواقع تعاني من شح الموارد. وفي حين أن العاملين في هكذا مواقع يقدمون في كثير من الأحيان رعايةً طبية رفيعة المستوى ويقومون بإجراء أنشطة البحوث الميدانية، لكنهم يعانون في العموم من قلة نصيبهم من الخبرة في إجراء التجارب السريرية المصممة للموافقة على الأدوية من جانب الجهات الرقابة الصارمة.

الأسلوب لقد قمنا بتنفيذ برنامج لبناء القدرات أثناء إجراء تجربة معشاة مضبطة بالشواهد متعددة المراكز لعقار "ديلامانيد"، وهو دواء جديد لعلاج مرض السل المقاوم للأدوية المتعددة. واشتمل البرنامج على ما يلي: (أ) تحديد الموقع وتقييم الاحتياجات، (ب) وتنفيذ المعايير الصادرة عن المؤتمر الدولي المعني بالتنسيق فيما يخص الممارسات السريرية الجيدة (ICH-GCP)، (جـ) وإرساء الأسس اللازمة لإدارة التجارب السريرية، (د) وزيادة المعرفة بالقضايا الرقابية العالمية والمحلية.

المواقع ****المحلية لقد تم إجراء التجارب في 17 موقعًا بتسع دول وهي: إستونيا، وبيرو، وجمهورية كوريا، والصين، والفلبين، ولاتفيا، ومصر، والولايات المتحدة الأمريكية

واليابان. وكان هناك ثمانية مواقع من إجمالي 10 مواقع شحيحة الموارد كانت تفتقر إلى عنصر الخبرة في تنفيذ التجارب السريرية اللازمة.

التغيّرات ****ذات ****الصلة تم إجراء عملية واسعة النطاق لبناء القدرات في المواقع العشرة جميعها. وقد أدى هذا البرنامج إلى تحسين القدرات المحلية في مجالات رئيسية مثل تصميم التجارب، وسلامة البيانات، والمراقبة، وتنفيذ التجارب، وخدمات المختبرات.

الدروس ****المستفادة لقد تبيّن أن التجارب السريرية المصممة لتوليد البيانات للحصول على الموافقة التنظيمية تتطلب بذل جهود إضافية لا تقف عند مجرد تعزيز القدرات البحثية التقليدية. وتوفر هذه الأساليب الرامية إلى بناء القدرات فرصةً لإقامة شراكات لتطوير المنتجات بغرض تحسين الأنظمة الصحية في المراحل اللاحقة على مجرد التنفيذ المباشر للتجربة السريرية المحددة.

## 摘要

**问题:**

在资源匮乏的地区,常常需要对感染性疾病的新药物进行评估。尽管在这些地区工作的人员提供高品质的服务并且开展业务研究活动,但是他们在执行供严格监管机构进行药物审批的临床试验方面往往经验不足。

**方法:**

我们在对新型耐多药结核病药物——delamanid 进行多中心随机对照试验期间,开展了一项能力建设项目。该项目包括:(1) 站点鉴定和需求评估;(2) 实现国际会议协调——良好药品临床试验规范 (ICH-GCP) 标准;(3) 创建试验管理;(4) 提升对全球及当地监管事项的了解。

**当地状况:**

试验在 9 个国家(埃及、爱沙尼亚、秘鲁、菲律宾、韩国、拉脱维亚、美国、日本和中国)的 17 个站点开展。其中 80% 的站点位于资源匮乏的地区,没有开展必要临床试验的经验。

**相关变化:**

10 个站点均完成了广泛的能力建设。该项目提高了当地在试验设计,数据安全和监控,试验开展和实验室服务等方面的能力。

**经验教训:**

旨在生成供监管机构审批数据的临床试验,在加强传统研究能力之外,还需要做出更多的努力。这种能力建设方法为产品开发合作伙伴关系提供了一个契机,在直接开展特定试验之外,改善卫生系统。

## Резюме

**Проблема:**

Новые лекарственные средства для лечения инфекционных заболеваний часто приходится оценивать в условиях нехватки ресурсов. Хотя люди в таких обстоятельствах часто обеспечивают уход высокого качества и проводят оперативные исследования, они, как правило, обладают меньшим опытом в области проведения клинических испытаний, разработанных для утверждения лекарственных средств в соответствии со строгими требованиями регулирующих органов.

**Подход:**

Программа по созданию потенциала была реализована во время многоцентрового рандомизированного контролируемого исследования деламанида — нового препарата для лечения туберкулеза со множественной лекарственной устойчивостью. Эта программа включала: (i) выявление места проведения исследования и оценку его потребностей; (ii) достижение соответствия стандартам Международной конференции по гармонизации и надлежащей клинической практике (ICH-GCP); (iii) внедрение управления исследованием; (iv) повышение осведомленности о мировых и местных вопросах нормативного регулирования.

**Местные условия:**

Исследования были проведены в 17 центрах 9 стран (Египет, Китай, Латвия, Перу, Республика Корея, Соединенные Штаты Америки, Филиппины, Эстония, Япония). Восемь из десяти центров, расположенных в странах с низким уровнем ресурсов, не имели опыта проведения требуемых клинических исследований.

**Осуществленные перемены:**

Во всех 10 центрах была проведена крупномасштабная работа по наращиванию потенциала для проведения исследований. В результате выполнения программы произошло укрепление местного потенциала в таких ключевых областях, как планирование хода исследования, защита данных и их мониторинг, проведение исследования и услуги лаборатории.

**Выводы:**

Клинические исследования, предназначенные для сбора данных, необходимых для получения законодательного разрешения, требуют совершения действий, выходящих за рамки традиционных мер по укреплению исследовательского потенциала. Такой подход к укреплению потенциала позволяет осуществлять партнерство с целью разработки продукции и улучшать системы здравоохранения для проведения более сложных мероприятий, чем отдельное исследование.

## Introduction

Traditional research capacity-building efforts tend to focus on post-approval clinical studies and operational research,^1^ rather than initial regulatory approval of new medicines.^2^^,^^3^ New medicines are needed for multidrug-resistant (MDR) tuberculosis^4^ and most of the people infected with MDR tuberculosis live in low-income countries, where there is often insufficient capacity to conduct clinical trials that meet the International Conference on Harmonization – Good Clinical Practice (ICH-GCP) standards.^5^^–^^8^ We describe a global clinical trial capacity-building programme done in the context of trials for delamanid conducted to achieve approval by a stringent regulatory authority.

## Programme design

The clinical development programme for delamanid was sponsored by Otsuka Pharmaceutical Development and Commercialization, Inc., conducted in partnership with national tuberculosis programmes and nongovernmental organizations. The programme consisted of three connected clinical trials: trial 204 was a three-month randomized, placebo-controlled trial (including a two-month hospitalization period).^9^ This was followed by trial 208, a six-month open-label extension of trial 204 in which participants had early access to delamanid.^10^ Finally, trial 116 followed all patients enrolled in trial 204 for 24 months. The trials were conducted from May 2008 to May 2012 at 17 sites in nine countries (China, Egypt, Estonia, Japan, Latvia, Peru, the Philippines, the Republic of Korea and the United States of America) with 481 participants completing trial 204 and 421 of these continuing into trial 116.

## Identification of programme sites

To identify and qualify clinical trial sites for participation in the delamanid programme, the partnership formed a multi-disciplinary site assessment team. The team consisted of experts in clinical trial management, public health and clinical aspects of MDR tuberculosis, including laboratory microbiology, diagnostics, data recording and reporting and disease management. The goals of the assessment team were to: (i) identify potential trial sites, mainly in low-resource settings; (ii) assess their capacity-building needs through a gap analysis; and (iii) gauge their potential to successfully conduct the delamanid trials with close long-term patient follow-up. The initial countries assessed (Estonia, Latvia, Peru and the Philippines) hosted the programmes used by the World Health Organization (WHO) to develop initial recommendations for the management of MDR tuberculosis.^11^ Additional sites were included to improve geographic diversity. Initial evaluation criteria were: access to MDR tuberculosis patients; experience with MDR tuberculosis management; and degree of compliance with ICH-GCP. Subsequently, each site was visited by trained clinical research associates from local contract research organization partners to identify specific capacity-building needs.

## Investing in key activities

To meet international standards for conducting clinical trials (i.e. ICH-GCP) several measures had to be taken. First, research personnel in 10 sites with limited experience in conducting such trials required assistance in procuring and maintaining additional equipment, direct interactions between sponsors and site staff and extensive oversight of trial monitoring activities. Up to 20% of all on-site monitoring visits were supervised by sponsor staff. These sites covered 90.4% (435/481) of participants in trial 204 and were located in Egypt, Estonia, Latvia, Peru, the Philippines and the Republic of Korea. Second, all sites required increased staff capacity and training (Table 1). Additional physicians, nurses and administrative personnel were hired so as not to disrupt routine clinical activity. To increase the number of trained professionals experienced both in tuberculosis treatment and in conducting clinical trials, sponsor-representatives trained all staff in ICH-GCP requirements. Training was first conducted at an investigator meeting where ICH-GCP and protocol requirements were reviewed in a lecture format. Subsequently, sponsor and contract research organization (CRO) representatives attended a site-specific initiation visit before each site enrolment. This visit summarized topics reviewed at the investigator meeting and provided more procedural detail on trial conduct. All site staff were required to attend the initiation visits and much of the training was interactive and focused on real-world patient scenarios. Throughout the conduct of the trial, 3–4 global team meetings were held with the entire sponsor and CRO teams. Research personnel were trained by the CRO partners or by sponsor representatives making site visits. The effectiveness of training was periodically reviewed throughout the trial by sponsor representatives reviewing all monitoring visit reports and attending 20% of the total monitoring visits at each site.

**Table 1:** Adaptations to site routines to comply with International Conference on Harmonization – Good Clinical Practice requirements when treating multidrug resistant tuberculosis

Practice	Site routines	ICH-GCP requirements	Measures to meet standards
Administration and storage of medication	Patients’ medications can be dispensed to a community health-care worker or relative on behalf of the patient	Patients’ medications must be dispensed only to the consented patient or maintained within appropriate storage at the clinical site	Increased capacity for DOT and drug storage at clinic or satellite clinic allowed patient to be dispensed medication directly
Directly-observed treatment	Family members or patient support staff can provide anti-tuberculosis drugs; patients may miss a small percentage of doses	Administration of drug under investigation must be conducted so that patients receive all doses directly from the health-care worker	When DOT not available, study medication dispensed for patients to take at home and held principal investigator to requirements of protocol
Adverse events	Common or mild adverse events associated with the standard MDR tuberculosis treatment may be omitted from patient charts	All adverse events must be recorded, regardless of their frequency, severity or causality	Provided extensive training for principal investigators and site staff. Increased monitoring and oversight frequency to ensure adverse events reporting requirements were met
Source data verification	Patient data is recorded in multiple locations, making the identification of an original source challenging	Identification of an original source for patients’ data is critical to the source data verification process	Developed source data templates and source data agreement with each site outlining where original data will be captured for all procedures
Patient confidentiality	Patient data might be recorded in log format, where patient anonymity is not guaranteed	Patient confidentiality is paramount, necessitating the creation of individual charts for trial participants	Provided staff ICH-GCP training and employed source data template

DOT: directly-observed treatment; ICH-GCP: International Conference on Harmonization – Good Clinical Practice; MDR: multidrug resistant; NTP: ….

Third, additional staff were hired to do laboratory procedures that were standardized with the use of a single laboratory manual. We improved hospital capacity by establishing better infection control measures and providing laboratory equipment and supplies. We invested in facility renovations and purchased equipment that was loaned to the site with an option to purchase at reduced cost once the trial was complete. All local staff were trained in the use of N95 or equivalent respirators and provided with essential personal equipment.

Finally, procurement of second-line drugs varied by location, but included a two-year course of second-line drugs for all participating patients. We ensured that specific second-line drugs required per protocol were obtained if they were locally unavailable and purchased the necessary storage equipment.

## Trial management

**Fig. 1:**
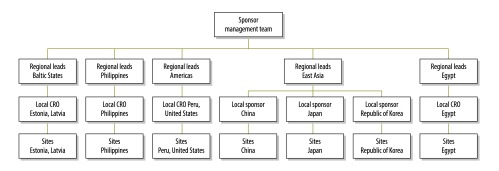
Management of the clinical development programme for a new tuberculosis medication, nine countries, 2008–2012

Staffing investments were made to ensure proper and consistent trial conduct according to ICH-GCP guidelines. The management structure was designed to allow consistent guidance and close oversight (Fig. 1). The trial management team was multidisciplinary and included representatives from the sponsor and local CROs. Sponsor team members were responsible for providing global oversight of the trial sites and direction to clinical research associates and personnel; face-to-face meetings were held with each clinical research associate throughout the trial. The training and re-training of CRO and site staff helped ensure comparability of data across countries. Each CRO was also assigned a regional lead person with extensive trial monitoring and project management expertise who was responsible for ensuring all operational requirements were met. Regional lead staff held bi-weekly teleconferences with their local counterparts throughout the trial. This approach fostered a higher level of communication between the trial management team and the local site staff than would have been possible with traditional outsourcing models.

CRO: contract research organization; MDR: multidrug resistant.

Note: The sponsor management team included specialists in MDR tuberculosis, clinical management and laboratory microbiology. Each geographical area was assigned two sponsor-based regional leads, specializing in clinical management and laboratory microbiology. Sponsor affiliates located in China, Japan and the Republic of Korea performed monitoring and site management responsibilities within these countries in lieu of the contract research organization.

## Regulatory processes

Three main regulatory issues needed to be considered: the protocol review and approval process, customs clearance and adherence to ICH-GCP standards. All sites required at least one institutional review board and government authority review but some settings required approvals from several groups. Often, these reviews needed to be conducted in series. The timelines for approval (from protocol submission for ethics review to final approval) for four countries comprising more than 75% of enrolled patients were as follows for trial 204: country 1, 8 weeks; country 2, 20 weeks; country 3, 41 weeks; and country 4, 32 weeks. Timelines were shorter for the second trial, due to acquired familiarity with regulatory processes and the existing approval of the first trial. Accordingly, the approval timelines for trial 208 were: country 1, 1 week; country 2, 9 weeks; country 3, 26 weeks; and country 4, 1 week. Import permits and customs clearance were straightforward given that all settings had existing regulations. Maintenance of ICH-GCP standards throughout the studies was ensured through a continuous independent auditing process.

## Lessons learnt

Several insights arise from our experience. First, sites reviewed and shown to meet international standards for MDR tuberculosis management were deemed well suited for clinical trials. Second, considerable capacity-building efforts were required, including training on the monitoring and management of adverse events, the maintenance of complete, accurate and confidential medical records and ensuring that all doses of study medication were taken (Table 1). Third, capacity-building activities helped address clinical and operational research priorities for drug-resistant tuberculosis, including validation of second-line drug testing, implementation and assessment of rapid diagnostic methods and implementation of monthly drug-susceptibility testing for optimization of treatment regimens.^12^ Fourth, institutional review board times were generally faster for the second trial, suggesting an advantage for conducting additional clinical trials in the same settings.

## Conclusion

Evaluating new products to meet regulatory approval standards requires additional efforts beyond traditional research capacity strengthening. Improving local capacity in key areas such as trial design, data safety and monitoring, trial conduct and laboratory services allows such settings to achieve ICH-GCP standards, to improve delivery of services to patients and establish a more permanent product evaluation infrastructure (Box 1). Indeed, all sites involved in this capacity-building programme are now equipped to assess new global health products targeting regulatory approval. However, it remains to be seen how these lessons can be translated on a wider scale. We suggest that private-sector partners, donors, governments and nongovernmental agencies create product evaluation centres-of-excellence. Such centres would generate local expertise in developing and evaluating products at all levels of pre-clinical and clinical development, with the intent of achieving approval by regulatory authorities. An array of stakeholders could ensure that global treatment priorities are being targeted. Pooled financing coupled with economies of scale would make such centres more financially feasible, potentially translating into reduced post-development prices.

Box 1 **Summary of main lessons learnt**

- Capacity-building activities differ between clinical trials designed to evaluate drugs for approval by regulatory authorities and clinical or operational research to improve care or develop health policy.
- When evaluating new drugs for tuberculosis, International Conference on Harmonization-Good Clinical Practice (ICH-GCP) standards may differ from WHO approaches recommended for national tuberculosis programmes; thus, additional efforts may be required to achieve the standards.
- Product development partnerships that improve local capacity in key areas such as trial design, data safety and monitoring, allow such settings to achieve ICH-GCP standards, improve delivery of services to patients and foster the ability to conduct future product evaluation trials in other therapeutic areas.

Building research capacity in low-resource settings is key for improving health systems and developing new medicines.^13^ Perceived challenges in successfully navigating research requirements in such settings often result in obstacles for product development partnerships.^14^^,^^15^ But as demonstrated here, incorporating appropriate capacity-building efforts into product development plans for novel therapeutics may offer a unique opportunity to reverse this trend and establish a long-term basis for similar future work. This includes using innovative approaches to evaluate drugs and optimize their use.^16^ The strategies described here for MDR tuberculosis drugs could serve as a practical roadmap for the development of high-quality clinical trial sites in low-resource settings.
